# Multiple Mechanisms of HIV-1 Resistance to PGT135 in a Chinese Subtype B’ Slow Progressor

**DOI:** 10.3390/pathogens14060556

**Published:** 2025-06-03

**Authors:** Yuanyuan Hu, Shasha Sun, Ying Liu, Li Ren, Xintao Hu, Yuhua Ruan, Liying Ma, Hao Liang, Yiming Shao, Kunxue Hong, Sen Zou, Yanling Hao

**Affiliations:** 1 Guangxi Key Laboratory of AIDS Prevention and Treatment & Biosafety III Laboratory, Guangxi Medical University, Nanning 530021, China; 2 National Key Laboratory of Intelligent Tracking and Forecasting for Infectious Diseases, National Center for AIDS/STD Control and Prevention, Chinese Center for Disease Control and Prevention, Beijing 102206, China; 3 NHC Key Laboratory of Biotechnology of Antibiotics, Institute of Medicinal Biotechnology, Chinese Academy of Medical Sciences & Peking Union Medical College, Beijing 100050, China

**Keywords:** HIV-1, bNAb, V3-glycan, pseudovirus, neutralization resistance, slow progressor

## Abstract

We investigated HIV-1 immune evasion mechanisms in a slow progressor (CBJC515) by constructing pseudoviruses expressing autologous Env proteins. Intriguingly, all pseudoviruses exhibited resistance to the broadly neutralizing antibody (bNAb) PGT135. Using site-directed mutagenesis and chimeric Env construction, we identified distinct escape mechanisms: early 2005 strains lost the N332 glycan site, while 2006/2008 strains retained key epitopes but developed resistance through structural modifications in the V1/V4/C2 regions or acquired novel N-glycosylation sites (N398/N611). These findings provide insights into how HIV-1 can escape from N332-directed bNAb responses without altering the epitope itself. Furthermore, chimeric experiments also elucidated regional co-evolution and functional maintenance: the V1V2 region broadly interfered with envelope protein function, while the V3 region may exhibit compensatory activity, restoring functionality and mitigating deleterious polymorphisms in other regions to keep Env antigenic diversity. These results offer valuable mechanistic clues that may inform the development of next-generation HIV-1 vaccines.

## 1. Introduction

Induction of broadly cross-neutralizing antibodies remains a critical yet unresolved challenge in HIV-1 vaccine development. While 10–30% of infected individuals naturally develop bNAbs after years of chronic infection [1,2,3,4], this demonstrates that bNAb induction is biologically possible. Promisingly, Haynes et al. [5] successfully induced polyclonal HIV-1 B cell lineages producing mature bNAbs by designing immunogens based on structural signatures of MPER-specific bNAbs, with the most potent antibodies neutralizing 15% of global tier-2 HIV-1 strains and 35% of clade B isolates. These breakthroughs highlight that focusing on structurally conserved functional epitopes—particularly those in key vulnerability regions—may be pivotal for effective bNAb induction.

Among these vulnerable targets, the V3-glycan supersites on HIV-1 Env represent key vulnerable regions targeted by bNAbs including PGT121, PGT128, and PGT135 [6,7]. Among these, the N332 glycan at the V3 loop base constitutes a major vulnerability site that enables broad antibody-mediated neutralization, making it a prime target for vaccine design [8,9]. While glycan loss at position 332 typically confers resistance to these bNAbs [10], numerous circulating strains retain this supersite yet remain resistant to neutralization [10,11]. Emerging evidence suggests that V1/V2 loop elongation may mediate this resistance [11,12,13,14,15], with van den Kerkhof et al. [11] demonstrating a statistically significant correlation between V1 length and PGT135 resistance. Nevertheless, the precise structural and functional determinants of this resistance remain unclear, particularly given the known disparities between structurally defined and functionally relevant epitopes [8,16]. Elucidating these resistance mechanisms could provide critical insights for engineering improved HIV-1 Env immunogens capable of eliciting more effective neutralizing antibody responses.

Furthermore, the HIV-1 gp120 envelope glycoprotein exhibits a sophisticated structural organization comprising five variable (V1–V5) and five constant (C1–C5) regions that collectively enable both immune evasion and functional preservation [17]. The surface-exposed variable regions undergo extensive sequence diversification to evade immune recognition [18,19,20], while the internal constant regions maintain structural integrity through a conserved core scaffold [21,22]. This delicate equilibrium is maintained through extensive co-evolutionary networks across gp120 domains, where mutations in one region are often compensated by changes in another to retain overall functionality [23,24]. Understanding these intricate relationships provides critical insights for engineering antigenic modifications that maintain native-like conformation while eliciting bNAb responses.

In this study, we generated CBJC515-derived Env-pseudotyped viruses and characterized their neutralization profiles against a panel of bNAbs (PGT121, VRC01, 12A21, 10E8, 2G12, and PGT135), revealing a striking universal resistance to PGT135 despite preserved sensitivity to other bNAbs. Through site-directed mutagenesis and chimeric Env construction, we identified three novel resistance mechanisms: (i) V1 loop elongation, (ii) V4/C2 structural change, and (iii) acquisition of N398/N611 glycosylation sites, demonstrating HIV-1’s capacity for immune evasion without altering core epitopes. Importantly, our domain-swapping experiments uncovered critical interdomain compensatory mechanisms, showing that while V1V2 diversification broadly modulates Env functionality, the V3 region serves as a key stabilizer that rescues structural defects induced by V1V2 or C2 modifications [25,26], providing fundamental insights for designing next-generation immunogens targeting conserved neutralization epitopes.

## 2. Materials and Methods

### 2.1. Study Subjects

The study subjects CBJC515 and CBJC437 were selected from a well-characterized Chinese cohort of chronic HIV-1 subtype B’ infections, distinguished by their plasma’s broad cross-neutralizing activity against a diverse panel of 25 viral strains [27]. These individuals acquired HIV-1 infection through commercial plasma donation between 1992–1995 and remained antiretroviral treatment (ART)-naïve throughout the study period. The major characteristics of CBJC515 are presented in Table 1; those of CBJC437 have been previously documented [28]. All study procedures were approved by the Institutional Review Board of the National Center for AIDS/STD Control and Prevention, China CDC, with written informed consent obtained from participants prior to blood collection and data acquisition.

### 2.2. Single-Genome Amplification and Env Clone Generation

Viral RNA was isolated from plasma samples using the QIAamp Viral RNA Mini Kit (Qiagen, Hessen, Germany), followed by immediate cDNA synthesis with the SuperScript III First-Strand Synthesis System (Invitrogen, Carlsbad, CA, USA). Full-length gp160 genes were amplified by single-genome amplification (SGA) as previously established [29]. Briefly, diluted cDNA was distributed into replicate PCR reactions in 96-well plates (Thermo Fisher Scientific, Waltham, MA, USA), with endpoint dilution performed to achieve a positivity rate below 30%, ensuring single-template amplification in most wells.

The SGA-derived PCR products were cloned into the vector pcDNA3.1D/V5-His-TOPO following the manufacturer’s instructions (Invitrogen, Carlsbad, CA, USA). The correct pcDNA3.1-Env plasmids used to produce pseudoviruses were selected by sequencing. First, the constructed plasmids were transformed into *E. coli* JM109 competent cells (Takara Bio, Shiga Prefecture, Japan), and then selecting monoclonal *E. coli* colonies on ampicillin-resistant LB medium (ampicillin concentration: 50 μg/mL) for sequencing. The verified correct *E. coli* clones were cultivated and then the plasmids were extracted using E.Z.N.A.^®^ Plasmid Mini Kit (Omega Bio-tek, Norcross, GA, USA) for pseudovirus production. The mutagenesis and chimeric plasmids described below, used to produce pseudoviruses, were selected as the same way.

### 2.3. Sequence Alignment and Bioinformatics Analysis

The SGA products were sequenced using an ABI 3700 Sequencer (Applied Biosciences, Foster City, CA, USA). Full-length *gp160* gene fragments were assembled and edited using Sequencher 4.1 (Gene Codes, Ann Arbor, MI, USA). All chromatograms were manually inspected for mixed bases (double peaks), and sequences exhibiting ambiguities were excluded from subsequent analyses. Env nucleotide sequences were aligned against the HIV-1 HXB2 reference strain using Gene Cutter (https://www.hiv.lanl.gov/content/sequence/GENE_CUTTER/cutter.html, last accessed 16 February 2025), and corresponding amino acid (aa) sequences were derived from the nucleotide alignments. Potential N-linked glycosylation sites (PNGS) were predicted using N-Glycosite (http://www.hiv.lanl.gov/content/sequence/GLYCOSITE/glycosite.html, last accessed 20 February 2025) from the Los Alamos HIV Database. Consensus sequences were generated using the online tool Consensus Maker (https://www.hiv.lanl.gov/content/sequence/CONSENSUS/consensus.html, last accessed 21 October 2020). Sequence identity and similarity between the 2005 Env consensus and individual functional clones were computed using the LALIGN tool (http://www.ch.embnet.org/software/LALIGN_form.html, last accessed 22 February 2025).

Additionally, a Chinese subtype B reference dataset (“B-Database”) was curated from the Los Alamos HIV Database (https://www.hiv.lanl.gov/components/sequence/HIV/search/search.html, last accessed 10 March 2025) by applying the following filters: *China*, *subtype B*, *intact gp120 sequences*, and *one sequence per patient*. This dataset comprised 168 high-quality sequences for comparative analysis. Phylogenetic analysis was performed using MEGA software (version 5.1).

### 2.4. Construction of Site-Directed Mutants and Chimeric Env Clones

Site-directed mutagenesis was performed using a standard PCR-based approach in a 50 μL reaction system containing 50 ng plasmid DNA template, 1 μL each of 10 μM forward (F) and reverse (R) primers (Sangon Biotech, Shanghai, China), 4 μL dNTP mixture (2.5 mM each) (Takara Bio, Shiga Prefecture, Japan), 25 μL 2×PrimeSTAR GC buffer (Takara Bio, Shiga Prefecture, Japan), 0.5 μL PrimeSTAR HS DNA polymerase (2.5 U/μL; Takara Bio, Shiga Prefecture, Japan), and nuclease-free water to volume. The mutagenic primers were designed to include the nucleotide sequences corresponding to five amino acid residues flanking both sides of the target mutation site, with the reverse primer being fully complementary to the forward primer. The thermal cycling protocol consisted of an initial denaturation at 98 °C for 1 min, followed by 30 cycles of denaturation (98 °C for 10 s), annealing (56 °C for 20 s; temperature optimized based on primer Tm values), and extension (72 °C for 6 min), with a final extension at 72 °C for 10 min. The complete Env gene of each resulting mutant was subsequently sequenced to verify the intended mutations and ensure no unintended changes were introduced.

For construction of chimeric clones, the GeneArt^®^ Seamless Cloning and Assembly Kit (Invitrogen, Carlsbad, CA, USA) was utilized to assemble PCR-linearized vector backbones with custom-synthesized DNA fragments (Sangon Biotech, Shanghai, China). The Env plasmid backbone was amplified using primers containing 15 bp overlaps complementary to the insertion sequences, followed by verification via 0.8% agarose gel (Addgene, Watertown, MA, USA) electrophoresis and purification using the QIAquick Gel Extraction Kit (Qiagen, Hessen, Germany). The assembled chimeric plasmids were rigorously validated through both full-length sequencing and restriction enzyme digestion analysis. Functional validation of mutants and chimeric clones was performed by preparing pseudoviruses and assessing their infectivity as follows. The modified regions in the chimeric constructs were precisely defined according to HXB2 gp160 reference positions: V1 (aa 131–157), V2 (aa 158–196), V1V2 (aa 131–196), C2 (aa 197–295), V3 (aa 296–331), V4 (aa 385–418), and V1–V3 (aa 131–331). These domain boundaries are consistent with established HIV-1 envelope protein topology [30], ensuring accurate domain swapping while maintaining structural integrity of the engineered Env proteins.

### 2.5. Pseudovirus Preparation and Titration

Pseudoviruses were generated by co-transfecting 4 × 10^6^ 293T/17 cells in a 75 cm² flask (Corning, New York, USA) with 5 μg Env expression plasmid (A260/A280 = 1.9) and 10 μg pSG3ΔEnv backbone vector using linear polyethylenimine (PEI Max, Polysciences, Warrington, PA, USA) at an N/P ratio of 7:1 (DNA:PEI ratio = 1:2 *w*/*w*). The PEI-DNA complexes were formed in serum-free DMEM (Life Technologies, Carlsbad, CA, USA) by incubating for 20 min at room temperature before addition to the cells. At 48 hours post-transfection, virus-containing supernatants were harvested, filtered through 0.45 μm membranes, and stored as 1 mL aliquots at −80 °C.

Pseudovirus infectivity was quantified using TZM-bl cells in a standardized TCID50 assay [28]. Each well of 96-well culture plates was filled with 100 μL DMEM medium, followed by the addition of 25 μL pseudovirus stock to the first column with thorough mixing. A 5-fold serial dilution was then performed by transferring 25 μL from column to column through column 11 (with final 25 μL discarded from column 11), while column 12 was maintained as virus-free cell control. This entire dilution series was prepared in quadruplicate. Then 1 × 10^4^ TZM-bl cells (containing DEAE-dextran, with a final concentration of 10 μg/mL) were added to each well and incubated for 48 hours in a cell culture incubator. Following incubation, 150 μL of supernatant was carefully removed from each well before adding 100 μL of Ultra-High Sensitivity Luminescence Reporter Gene Assay System (PerkinElmer, Waltham, MA, USA). After complete cell lysis, lysates were transferred to a black 96-well plate for luminescence measurement. Wells demonstrating relative luminescence units (RLUs) exceeding three-fold background (cell control) levels were considered positive for infection. The TCID_50_ titers were determined using four independent replicates for each virus dilution series, with each replicate including technical duplicates. The final TCID_50_ values represent the geometric mean (±SD) across these replicates, calculated using the Reed–Muench method implemented in the TCID_50_ Macro for Microsoft Excel, provided by the Comprehensive Antibody-Vaccine Immune Monitoring Consortium (CAVIMC). This standardized computational tool ensures reproducible quantification of viral infectivity.

### 2.6. Neutralization Assay

The neutralization activity was assessed by measuring the reduction in luciferase reporter gene expression following a single-round infection of TZM-bl cells, as previously described [27]. Pseudoviruses were normalized to 200 TCID_50_, and 50 μL aliquots were incubated with 100 μL of threefold serially diluted bNAbs (in duplicate) for 1 h at 37 °C in 96-well flat-bottom plates (Corning, New York, NY, USA). The bNAb starting concentration was 20 μg/mL. Subsequently, the virus-antibody mixtures were used to infect 1 × 10^4^ TZM-bl cells (100 μL/well) in the presence of DEAE-dextran (final concentration: 10 μg/mL). The 96-well plate was prepared with two control groups: column 1 served as a cell control with 150 μL DMEM and 100 μL TZM cells per well, while column 2 functioned as a virus control containing 100 μL DMEM, 50 μL viral suspension, and 100 μL TZM cells in each well. Following 48 h of incubation, infection levels were quantified by measuring luciferase activity. For luminescence measurement, 150 μL of culture supernatant was carefully removed from each well, and 100 μL of Ultra-High Sensitivity Luminescence Reporter Gene Assay System was added for 2 min to achieve complete cell lysis. Then, a 150 μL aliquot of each lysate was transferred to a black 96-well plate, and luminescence was measured using a PerkinElmer luminometer (PerkinElmer, Waltham, MA, USA). The 50% inhibitory dose was calculated as the antibody concentration that reduced relative luminescence units (RLU) by 50% compared to virus control wells (set as 100% infection).

### 2.7. Neutralizing Antibodies and Cell Lines

The bNAbs PGT135, PGT121, 2G12, 12A21, 10E8, and VRC01 were generously provided by the NIH AIDS Research and Reference Reagent Program. 293T/17 cells were purchased from the American Type Culture Collection (catalog no. 11268). The 293T/17 cell line is a derivative strain capable of generating high-titer infectious retroviruses. TZM-bl cells were obtained from the NIH AIDS Research and Reference Reagent Program (ARRRP, catalog no. 8129).

### 2.8. Statistical Analyses

GraphPad Prism 9.0 was used to perform statistical analyses and graphical representation. The IC_50_ of the antibody was calculated in GraphPad Prism by fitting a dose–response curve with a four-parameter logistic model, using nonlinear regression to determine the antibody concentration that inhibits 50% of viral activity.

## 3. Results

### 3.1. Neutralization Sensitivity Profiles of CBJC515 HIV-1 Strains Against bNAbs

From longitudinal CBJC515 plasma samples collected in August 2005, April 2006, and November 2008, we successfully generated 6, 3, and 11 functional pseudovirus clones, which were designated as the CBJC515-2005, CBJC515-2006, and CBJC515-2008 isolates, respectively. Neutralization profiling against prototypic bNAbs (PGT121, VRC01, 12A21, 10E8, 2G12, and PGT135) revealed that all 20 clones maintained sensitivity to CD4 binding site-specific bNAbs (VRC01 and 12A21) and the MPER-targeting bNAb 10E8 (Table 2). Notably, while all isolates showed sensitivity to PGT121, they exhibited complete resistance to PGT135, both of which target the N332 glycan supersite [6,31]. Building on these findings, we performed phylogenetic analysis to determine the distribution patterns of the 20 clones among representative global HIV reference strains (Figure S1) and the Chinese subtype B reference dataset (Figure S2).

### 3.2. Lack of 332 Glycosylation Site Leads to Resistance to PGT135 in CBJC515-2005 Isolates

The universal resistance of CBJC515-derived clones to the V3-glycan targeting bNAb PGT135 prompted detailed investigation of its underlying mechanisms. PGT135 mediates neutralization through interactions with glycans at Asn332, Asn386, and Asn392, utilizing extended CDR H1 and H3 loops to penetrate the glycan shield and access conserved gp120 epitopes [6]. We first examined the N332 position in these strains. As shown in Table 3, the N332 glycan site was absent in all CBJC515-2005 Env clones but present in all CBJC515-2006/2008 clones. Specifically, clone CBJC515-2005-8 exhibited a D332 substitution (HXB2 numbering), while other CBJC515-2005 variants possessed an N334 glycan that precluded N332 glycosylation. We conducted site-directed mutagenesis experiments, which introduced specific amino acid substitutions into viral proteins to evaluate their impact on antibody neutralization. We constructed six point-mutated pseudoviruses of CBJC515-2005 strains and detected their neutralizing sensitivity to PGT135. The results confirmed the critical role of this site, as D332N or N334S substitutions restored sensitivity to both PGT135 and 2G12 in all CBJC515-2005 variants (Table 4). However, additional mutations targeting reported PGT135 contact residues (R/K389Q, T409E, Y330H) [6,12,32] failed to alter neutralization profiles (Table 3), suggesting these residues may not contribute significantly to the observed resistance phenotype in these strains.

In our study, CBJC515-2005 pseudoviruses bearing the N334 glycan site were completely resistant to neutralization by PGT135 and 2G12, yet remained fully sensitive to PGT121. These findings align with previous reports showing that while some N332-dependent bNAbs can accommodate glycan shifts to N334, others exhibit strict specificity for the canonical N332 site [6,10].

### 3.3. Chimeric Analysis Reveals Region-Specific Effects on Neutralization Sensitivity in CBJC515-2006/2008 Isolates

To investigate the determinants of neutralization resistance in CBJC515-2006/2008 strains harboring key epitopes (Table 3), we performed sequence analysis and fragment chimerism experiments. The fragment chimerism experiment constructed chimeric viruses by swapping fragments between different viral strains, playing an essential role in dissecting the mechanisms of viral resistance to neutralizing antibodies. This approach allows researchers to pinpoint exact viral regions responsible for antibody evasion by systematically testing how fragment exchanges alter neutralization sensitivity. The CBJC515-2005 isolates exhibited a shorter, more conserved V1 region (22 aa) (Figure 2), whereas the CBJC515-2006/2008 strains displayed significant V1 expansion (22–42 aa), exceeding the length of the HIV-1 subtype B consensus sequence (25 aa) [14]. To assess whether this elongation conferred resistance to PGT135, we replaced the V1 region (positions 131—157) in CBJC515-2006/2008 Env clones with that of CBJC515-2005-8 (D332N)—selected as the most representative CBJC515-2005 strain based on sequence harmony (SH) analysis relative to the CBJC515-2005 Env consensus sequence (Table 5).

Sequence ‘% identity’ reflects exact amino acid matches in ungapped alignments, while ‘% similarity’ includes conserved substitutions with comparable biochemical properties (e.g., charge or hydrophobicity).

**Figure 1:**
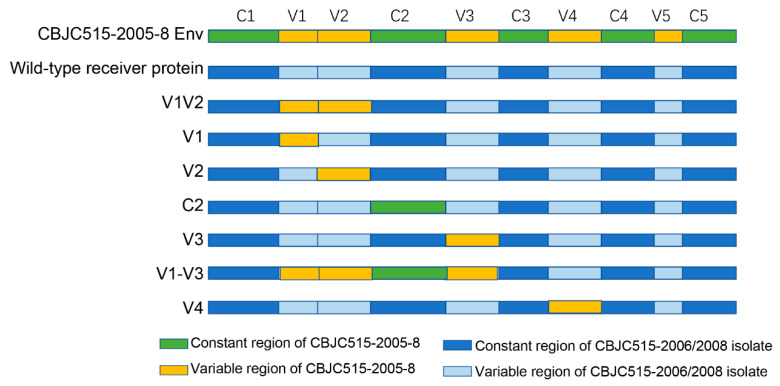
Schematic representation of chimeric HIV-1 Env constructs. Donor (CBJC515-2005-8) Gp120: C1–C5 (green), V1–V5 (yellow). Recipient (wild-type receiver protein, CBJC515-2006/2008) backbone: C1–C5 (dark blue), V1–V5 (light blue). All domains follow HXB2 numbering.

**Figure 2:**
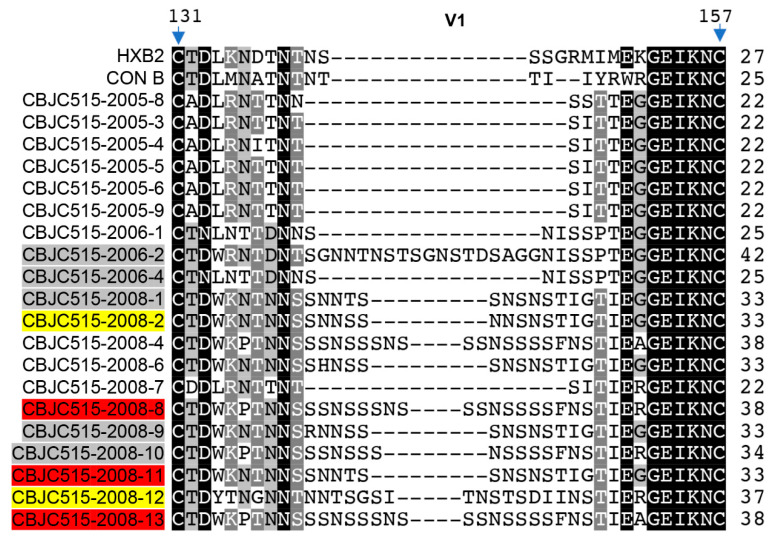
Alignment of the V1 region sequences. The HIV-1 subtype B’ consensus sequence (CON B) is shown as a reference. Amino acid positions correspond to HXB2 residues 131–157. Sequence conservation is indicated by grayscale shading (gray, dark gray, and black for increasingly conserved residues). Gaps are denoted by dashes (–). The right column indicates V1 region length. Chimera names are color-coded by phenotype: red (sensitive), yellow (resistant), and gray (nonfunctional).

Eleven V1 chimeras were successfully constructed, comprising two strains derived from 2006 isolates and nine strains based on 2008 variants (Figure 1 and Figure 2, Table 6). Six chimeras (wild-type V1 lengths ranging from 22 to 42 aa) failed to produce infectious clones (two strains derived from 2006 isolates and four strains derived from 2008 variants) (Table 6). Two chimeras, CBJC515-2008-2 (2005-8 V1) and CBJC515-2008-12 (2005-8 V1), with original V1 lengths of 33 and 37 aa, respectively, maintained PGT135 resistance, while three—CBJC515-2008-8 (2005-8 V1), CBJC515-2008-11 (2005-8 V1), and CBJC515-2008-13 (2005-8 V1)—became PGT135-sensitive, showing IC_50_ values of 1.19 μg/mL, 5.8 μg/mL, and 8.3 μg/mL, respectively.

Given that the V1V2 region globally modulates neutralization sensitivity [30,33,34], and that prior studies linked V3-glycan bNAb efficacy to V1V2 length in the presence of the N332 glycan site [12,35], we constructed seven V1V2 chimeras by replacing the CBJC515-2006/2008 V1V2 region with that of CBJC515-2005-8, including one strain from 2006 and six strains derived from 2008 (Table 6). Neutralization assays revealed that five chimeras were nonfunctional, while CBJC515-2008-2 (2005-8 V1V2) and CBJC515-2008-12 (2005-8 V1V2) remained functional and resistant. Additionally, among the three V2 chimeras that we constructed (derived from 2008), only one retained functionality and resistance, suggesting that the V1 loop is the primary determinant of resistance within the V1V2 region (Table 6).

**Figure 3:**
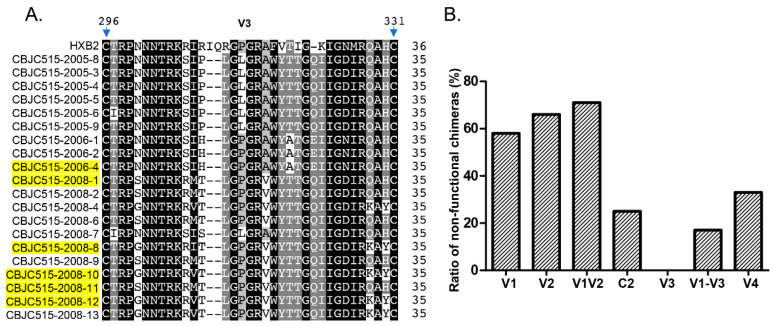
Sequence alignment of the V3 region (**A**) and frequency of nonfunctional chimeras with different envelope regions (**B**). (**A**) The V3 region alignment shows conserved amino acid sequences represented by grayscale shading (gray, dark gray, and black for increasing conservation). Resistant chimeras are highlighted in yellow. (**B**) The bar graph illustrates the proportion of nonfunctional chimeras generated for each chimeric region (V1, V2, V1V2, C2, V3, V1-V3, and V4).

To examine whether other genomic regions influence PGT135 sensitivity during viral evolution (CBJC515-2006/2008 timepoints), we generated chimeric envelopes by replacing C2, V3, V1-V3, or V4 regions in CBJC515-2006/2008 isolates with their CBJC515-2005-8 (D332N) counterparts (Table 7, Figure 1). We constructed a total of 19 chimeric pseudoviruses, including four C2-region chimeras (comprising one strain isolated in 2006 and three strains from 2008), three V4-region chimeras (containing one 2006 strain and two 2008 strains), and twelve V1-V3-region chimeras (comprising two strains isolated in 2006 and 10 strains from 2008). Neutralization assays revealed that (i) all six V3 chimeras maintained infectivity and sensitivity (Figure 3); (ii) three of four C2 chimeras remained functional (one became sensitive); (iii) ten of twelve V1–V3 chimeras were functional (three sensitive); and (iv) two of three V4 chimeras were functional (both sensitive).

### 3.4. N611A and N398A Glycan Mutations Alter Neutralization Sensitivity to PGT135

**Figure 4:**
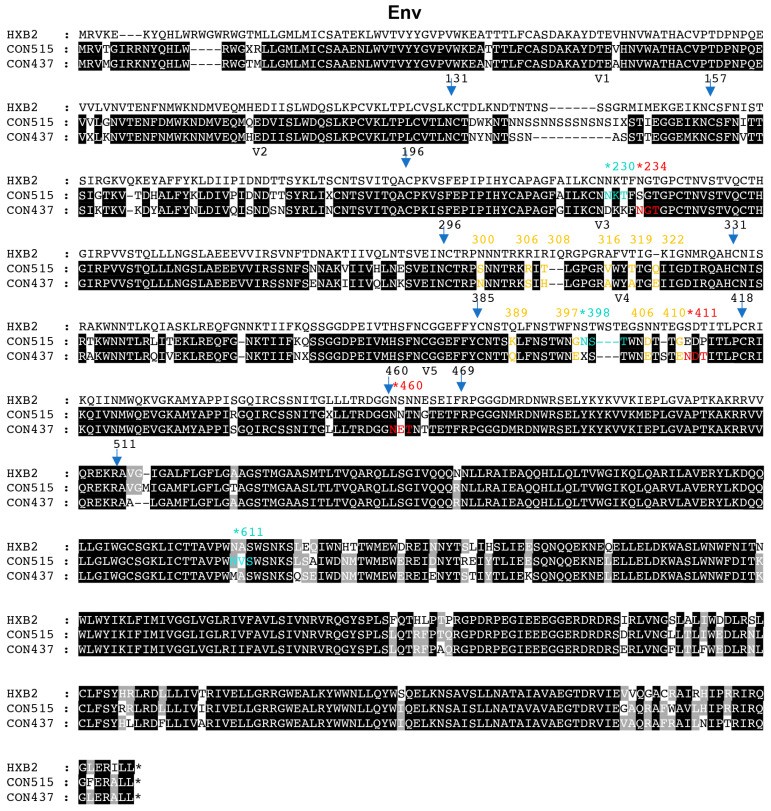
Alignment of consensus sequences between CBJC515 and CBJC437 strains. Potential N-linked glycosylation sites are marked with asterisked numbers. Blue and red fonts indicate CBJC515-specific and CBJC437-specific glycosylation sites (V1 region excluded), respectively, as predicted by N-Glycosite analysis. Yellow fonts highlight non-glycosylated polymorphic sites in the V3 and V4 regions. Arrows with adjacent numbers demarcate the boundaries of variable (V) and constant (C) regions.

To further investigate resistance mechanisms in CBJC515-2006/2008 isolates, we analyzed CBJC437, a Chinese subtype B´ slow progressor whose isolates contained key epitopes (N332/N386/N392/N295/H330) and remained PGT135-sensitive (Table S1). By comparing the consensus sequences of CBJC515 and CBJC437 (Figure 4), we found that the CBJC515 gp160 protein contains 28 glycosylation sites, with 23 located on gp120, while the CBJC437 gp160 protein has 31 glycosylation sites, 27 of which are on gp120. Alignment of glycosylation site positions revealed that most sites overlap between the two consensus sequences. Further analysis of each gp160 sequence (excluding the V1 region, as its sequence exhibits significant variability and may adversely affect viral activity) identified N230, N398, and N611 as CBJC515-specific glycosylation sites, whereas N234, N405, and N411 were unique to CBJC437. Through site-directed mutagenesis, we sequentially eliminated the CBJC437-specific glycosylation sites (N234, N405, N411), which resulted in no observable alteration in PGT135 neutralization sensitivity (Table S2). Similarly, when we eliminated the CBJC515-specific glycosylation sites (N230, N398, and N611), we observed that two mutations demonstrated strain-specific restoration of PGT135 sensitivity: N398A in CBJC515-2006-4/ CBJC515-2008-13 (IC_50_: 7.2 μg/mL and 6.9 μg/mL, respectively) and N611A in CBJC515-2008-9 (IC_50_: 2.6 μg/mL) (Table S3).

### 3.5. Statistical Analysis of PGT135-Targeted Glycosylation Sites in Chinese HIV-1 Subtype B’ Strains

Previous studies indicate that PGT135 exhibits a neutralization breadth of approximately 33%, comparable to the CD4bs-class antibody b12 but notably lower than other N332-dependent antibodies such as PGT121 and PGT128 [7,31]. This restricted neutralization capacity has been primarily attributed to the limited prevalence of critical contact residues (Asn332, Asn392, and His330) in circulating HIV-1 strains [6].

Our analysis of 168 Chinese subtype B´ sequences from the Los Alamos HIV database (B-Database) revealed that the essential PGT135 epitopes (H330, N332, N392) were present in 76% (127/168) of strains. Furthermore, when considering the strain-dependent recognition of N295/N386 glycans [6], we found that 66% (111/168) and 67% (112/168) of sequences contained (H330+N332+N392) plus either N295 or N386, respectively. Strikingly, all four glycosylation sites (N332+N392+N295+N386) were present in 60% (100/168) of sequences, while the N332 site alone appeared in 93.5% (157/168) of strains. These prevalence rates significantly exceed PGT135’s observed 33% neutralization breadth, strongly suggesting that many Chinese HIV-1 subtype B´ strains containing PGT135 epitopes have evolved effective resistance mechanisms, warranting investigation into these evasion strategies.

## 4. Discussion

The induction of bNAb responses remains a central challenge in HIV vaccine development. Understanding escape mechanisms in broadly cross-neutralizing samples could provide critical insights for immunogen design [15,36,37,38]. Our study reveals multiple unconventional pathways of PGT135 escape, with the most significant novelty being the demonstration of epitope-independent resistance mechanisms in HIV-1. While canonical escape via N332 glycan loss was observed in early strains (2005), later strains (2006/2008) evolved resistance while preserving all critical PGT135 contact residues (N332/N386/N392/H330). This challenges the prevailing paradigm that bNAb escape necessarily requires epitope alteration and establishes three previously unrecognized evasion strategies: (1) V1 loop elongation, (2) V4/C2 structural change, and (3) acquisition of N398/N611 glycosylation sites. While previous work hypothesized that V1 elongation mediates escape [11], we experimentally demonstrated that V1 replacement can restore sensitivity in some strains, whereas V1V2 or V2 substitutions cannot. These findings, combined with prior studies on PGT121 and PGT128 [10,13], underscore the broad impact of V1 length on V3-glycan bNAb neutralization, likely by sterically hindering access to the V3-glycan supersite [15,39]. We also identified additional roles for V4 and C2 regions in neutralization resistance. Through extensive mutagenesis, we found that single amino acid changes rarely restore sensitivity, suggesting that extreme variations may be required for PGT135 escape in N332^+^ strains [14]. The N398 residue is positioned within the V4 region, which has been implicated in modulating viral neutralization sensitivity [6]. N611 is situated within the gp41 region, proximal to the fusion peptide of gp41. Studies indicate that N611 serves as a critical neutralizing epitope, and the N611A mutation has been shown to markedly enhance antibody neutralization efficacy [40]. However, the mechanistic impact of the N398A and N611A mutations on the neutralization capacity of PGT135 remains unclear and warrants further investigation.

In donor CBJC515, we observed dynamic evolution of PNGS patterns, with N332 glycosylation sites emerging and becoming fixed by the 2006/2008 timepoints. While the N332 mutation in CBJC515-2005 strains represents the canonical escape route from V3-glycan bNAbs [10], the persistence of this epitope in later strains suggests competing selection pressures from strain-specific NAbs or other antibody lineages [15,36]. This may have driven the virus to adopt alternative escape strategies while maintaining the N332 site. The fluctuating neutralization breadth from 2005–2009 (Table 1) and evidence of coexisting antibody lineages [41] support this hypothesis. In natural HIV-1 infection, the development of bNAbs typically requires several years of viral evolution and antigen exposure [37,42,43]. This prolonged maturation process reflects both the need for extensive antibody somatic hypermutation and the complex structural features of conserved envelope epitopes that must be recognized. The retention of N332 through unconventional escape mechanisms may prolong epitope exposure, potentially facilitating bNAb maturation [15,36,37,38], as suggested by our observations of incomplete viral escape (data not shown). Further investigation through plasma epitope mapping and B-cell sequencing could elucidate these unusual escape patterns.

Our study reveals how HIV-1 maintains the delicate balance between immune evasion and viral fitness through coordinated evolution of envelope protein domains. While gp120’s hypervariable regions (particularly V1V2) permit extensive sequence diversification, our chimeric experiments demonstrate their functional dependence on co-evolved structural partners—evidenced by loss of Env functionality in 5/7 V1V2, 6/11 V1, and 2/3 V2 replacements (Table 6, Figure 3B). Similar constraints were observed in V4 (1/3 nonfunctional) and C2 (1/4 nonfunctional) chimeras (Table 7, Figure 3B), highlighting how HIV-1 preserves infectivity through precisely coordinated variations across structurally linked regions. These findings provide experimental validation of the “constrained diversification” model [25,26], wherein the virus accumulates immune-escape mutations while maintaining critical functional networks through compensatory coevolution.

Consistent with previous reports [26], our chimeric studies demonstrated that V3 region replacement had minimal impact on Env functionality, with all V3-modified clones remaining functional (Figure 3A). This remarkable tolerance to substitution likely stems from the V3 loop’s high conservation, which preserves the structural integrity of the co-receptor binding site [44]. Notably, when we replaced the entire V1-V3 region (encompassing V1, V2, C2, and V3 domains), only 2 of 12 chimeras lost functionality (Figure 3B), a significantly lower failure rate than observed with individual V1, V2, or C2 replacements. This finding suggests both coordinated evolution among these regions and a unique capacity of V3 to facilitate functional recovery. A striking example was observed in the CBJC515-2006-4 strain: while individual V1V2 or C2 replacements abrogated function, the complete V1-V3 chimera restored viability. These results align with recent studies demonstrating V3’s role in maintaining genetic robustness by compensating for structural perturbations [25,45].

Our findings significantly advance the understanding of PGT135 resistance mechanisms, revealing that even strains retaining key epitopes can evade neutralization through diverse modifications—including V1/V4/C2 alterations and N398/N611 glycosylation. These unconventional escape routes offer two key translational implications: (1) regarding vaccine design, it necessitates targeting conserved envelope regions beyond the primary epitope to block compensatory structural adaptations; (2) regarding antibody therapy, it demands combinatorial approaches that simultaneously address epitope variation and conformational shielding.

Additionally, molecular dynamics (MD) simulations have been widely employed to investigate protein-protein interactions and can be effectively applied to study the evolutionary mechanisms of viral membrane proteins. For instance, Han et al. utilized Gaussian accelerated molecular dynamics (GaMD) simulations combined with Markov state models to elucidate the inhibitory mechanism of fullerene-linear polyglycerol-b-aminosulfate (F-LGPS) on the spike protein [46]. Similarly, Yu et al. demonstrated through homology modeling that VMP-63 and VMP-108 exhibit superior binding affinity with CBFβ [47]. In future studies, we plan to enhance our investigations of interprotein molecular dynamics simulations to provide more comprehensive theoretical insights into viral evolutionary mechanisms.

## Figures and Tables

**Table 1:** The profile of the study subject CBJC515.

Sample Date	No. of CD4^+^ T cells/μL	No. of Viral RNA Copies/mL	Geometric Mean ID_50_ Titres	No. of Functional Clones	Breath
20050816	528	1.94 × 10^4^	−	6	−
20051101	530	6.94 × 10^3^	61.7	−	77.3%
20060418	536	1.55 × 10^4^	161.6	3	90.9%
20061123	476	2.90 × 10^4^	203.0	−	95.5%
20070424	543	6.69 × 10^3^	106.0	−	81.8%
20080415	459	2.22 × 10^4^	−	−	−
20081118	321	1.25 × 10^5^	94.1	11	86.4%
20090519	268	8.56 × 10^4^	164.8	−	95.5%

‘−’ indicates no data were available.

**Table 2:** Neutralization profiling of CBJC515 pseudoviruses against bNAbs.

Pseudoviruses	IC_50_ (μg/mL)					
	PGT121	VRC01	12A21	10E8	2G12	PGT135
CBJC515-2005-3	0.7	0.1	>10	1.0	>10	>10
CBJC515-2005-4	0.8	0.2	>10	0.2	>10	>10
CBJC515-2005-5	1.3	0.1	0.1	0.3	>10	>10
CBJC515-2005-6	1.8	0.1	0.1	0.3	>10	>10
CBJC515-2005-8	0.8	0.1	>10	0.3	>10	>10
CBJC515-2005-9	1.8	0.2	>10	0.4	>10	>10
CBJC515-2006-1	1.2	4.8	4.1	3.1	0.6	>10
CBJC515-2006-2	5.8	2.1	1.9	0.7	0.9	>10
CBJC515-2006-4	0.5	1.9	1.1	1.2	0.5	>10
CBJC515-2008-1	0.2	3.5	2.6	0.9	0.7	>10
CBJC515-2008-2	0.8	4.2	3.1	2.2	0.8	>10
CBJC515-2008-4	6.7	6.2	0.4	0.9	3.4	>10
CBJC515-2008-6	0.7	3.5	3.1	0.1	0.7	>10
CBJC515-2008-7	2.1	0.1	5.3	0.3	>10	>10
CBJC515-2008-8	0.3	4.8	0.4	0.4	5.4	>10
CBJC515-2008-9	0.5	3.1	2.0	0.9	0.6	>10
CBJC515-2008-10	1.1	3.8	0.1	0.3	0.9	>10
CBJC515-2008-11	0.5	4.3	2.3	0.5	0.5	>10
CBJC515-2008-12	3.0	2.3	1.3	0.7	2.4	>10
CBJC515-2008-13	0.2	2.8	0.7	0.7	0.5	>10

‘>10’ indicates the pseudovirus resistance to the bNAb; ‘CBJC515’: sample ID; ‘2005’ indicates the collection date, and the same applies to 2006 and 2008.

**Table 3:** The characterization of PGT135 epitopes in CBJC515 HIV-1 Env clones.

Env Clones	N-Glycan Site				Q389	E409	H330
	332	295	386	392			
CBJC515-2005-3	−	+	+	+	R	T	.
CBJC515-2005-4	−	+	+	+	R	T	.
CBJC515-2005-5	−	+	+	+	R	T	.
CBJC515-2005-6	−	+	+	+	R	T	.
CBJC515-2005-8	−	+	+	+	R	T	.
CBJC515-2005-9	−	+	+	+	R	T	.
CBJC515-2006-1	+	+	+	+	K	T	.
CBJC515-2006-2	+	+	+	+	K	T	.
CBJC515-2006-4	+	+	+	+	K	T	.
CBJC515-2008-1	+	+	+	+	K	T	.
CBJC515-2008-2	+	+	+	+	K	T	.
CBJC515-2008-4	+	+	+	+	K	T	Y
CBJC515-2008-6	+	+	+	+	K	T	.
CBJC515-2008-7	+	+	+	+	R	T	.
CBJC515-2008-8	+	+	+	+	K	T	Y
CBJC515-2008-9	+	+	+	+	K	T	.
CBJC515-2008-10	+	+	+	+	K	T	Y
CBJC515-2008-11	+	+	+	+	K	T	.
CBJC515-2008-12	+	+	+	+	K	T	Y
CBJC515-2008-13	+	+	+	+	K	T	Y

‘**+**’ = Presence of a potential N-linked glycosylation site (PNGS) at the indicated position; ‘**−**’ = Absence of a PNGS at the corresponding position; ‘**.**’ = Amino acid matches the consensus epitope residue.

**Table 4:** The neutralizing sensitivity of mutated pseudoviruses to PGT135.

Mutated Pseudoviruses	IC_50_ (μg/mL)	
	PGT135	2G12
CBJC515-2005-8 (D332N)	0.6	1.8
CBJC515-2005-3 (N334S)	6.7	0.8
CBJC515-2005-4 (N334S)	1.5	1.9
CBJC515-2005-5 (N334S)	0.7	1.0
CBJC515-2005-6 (N334S)	5.1	>10
CBJC515-2005-9 (N334S)	0.9	1.7

‘>10’ indicates the pseudovirus resistance to PGT135.

**Table 5:** Sequence similarity analysis of 2005 functional Env variants relative to the 2005 consensus Env.

CBJC515-2005 Envs	% Identity	% Similarity
Con CBJC515-2005	100.0	100.0
CBJC515-2005-3	98.0	99.5
CBJC515-2005-8	99.5	99.6
CBJC515-2005-4	99.3	99.5
CBJC515-2005-5	99.3	99.5
CBJC515-2005-6	98.5	99.3
CBJC515-2005-9	98.7	99.5

‘Con CBJC515-2005’: The consensus sequence of CBJC515 Env sequences from 2005.

**Table 6:** Neutralization sensitivity of CBJC515 V1/V1V2/V2 chimeric pseudoviruses to PGT135.

Chimeric Pseudoviruses	IC_50_ (μg/mL)
V1 chimeric pseudoviruses	
CBJC515-2006-2 (2005-8 V1)	−
CBJC515-2006-4 (2005-8 V1)	−
CBJC515-2008-1 (2005-8 V1)	−
CBJC515-2008-2 (2005-8 V1)	>10
CBJC515-2008-7 (2005-8 V1)	−
CBJC515-2008-8 (2005-8 V1)	1.2
CBJC515-2008-9 (2005-8 V1)	−
CBJC515-2008-10 (2005-8 V1)	−
CBJC515-2008-11 (2005-8 V1)	5.8
CBJC515-2008-12 (2005-8 V1)	>10
CBJC515-2008-13 (2005-8 V1)	8.3
V1V2 chimeric pseudoviruses	
CBJC515-2006-4 (2005-8 V1V2)	−
CBJC515-2008-1 (2005-8 V1V2)	−
CBJC515-2008-2 (2005-8 V1V2)	>10
CBJC515-2008-8 (2005-8 V1V2)	−
CBJC515-2008-10 (2005-8 V1V2)	−
CBJC515-2008-11 (2005-8 V1V2)	−
CBJC515-2008-12 (2005-8 V1V2)	>10
V2 chimeric pseudoviruses	
CBJC515-2008-8 (2005-8 V2)	−
CBJC515-2008-10 (2005-8 V2)	−
CBJC515-2008-12 (2005-8 V2)	>10

‘−’: Nonfunctional chimeric pseudovirus; ‘>10’ indicates chimeric pseudovirus resistance to PGT135.

**Table 7:** Neutralizion sensitivity of CBJC515 C2/V4/V1-V3 chimeric pseudoviruses to PGT135.

Chimeric Pseudoviruses	IC_50_ (μg/mL)
C2 chimeric pseudoviruses	
CBJC515-2006-4 (2005-8 C2)	−
CBJC515-2008-4 (2005-8 C2)	8.3
CBJC515-2008-10 (2005-8 C2)	>10
CBJC515-2008-12 (2005-8 C2)	>10
V4 chimeric pseudoviruses	
CBJC515-2006-1 (2005-8 V4)	2.7
CBJC515-2008-2 (2005-8 V4)	−
CBJC515-2008-6 (2005-8 V4)	0.3
V1-V3 chimeric pseudoviruses	
CBJC515-2006-1 (2005-8 V1-V3)	>10
CBJC515-2006-4 (2005-8 V1-V3)	>10
CBJC515-2008-1 (2005-8 V1-V3)	−
CBJC515-2008-2 (2005-8 V1-V3)	>10
CBJC515-2008-4 (2005-8 V1-V3)	>10
CBJC515-2008-6 (2005-8 V1-V3)	>10
CBJC515-2008-8 (2005-8 V1-V3)	4.8
CBJC515-2008-9 (2005-8 V1-V3)	>10
CBJC515-2008-10 (2005-8 V1-V3)	2.6
CBJC515-2008-11 (2005-8 V1-V3)	−
CBJC515-2008-12 (2005-8 V1-V3)	1.2
CBJC515-2008-13 (2005-8 V1-V3)	>10

‘−’: Nonfunctional chimeric pseudovirus; ‘>10’ indicates chimeric pseudovirus resistance to PGT135.

## Data Availability

The data presented in this study are available in this published article. Further inquiries can be directed to the corresponding author.

## Supplementary Materials

The following supporting information can be downloaded at: https://www.mdpi.com/article/10.3390/pathogens14060556/s1; Table S1: Neutralization sensitivity of CBJC437 clones to PGT135. Table S2: The neutralization sensitivity of CBJC437 mutations to PGT135. Table S3: The neutralization sensitivity of CBJC515 mutations to PGT135. Figure S1. The neighbor-joining phylogenetic tree showing the placement of 20 clones within global HIV reference strains. Figure S2. The neighbor-joining phylogenetic tree illustrating the distribution of 20 clones within the Chinese subtype B reference dataset.
