# Supplementary material for: Multiple Mechanisms of HIV-1 Resistance to PGT135 in a Chinese Subtype B’ Slow Progressor
Source: Pathogens. 2025 Jun 3;14(6):556. doi: 10.3390/pathogens14060556 (PMC12196039; doi:10.3390/pathogens14060556)
Supplement: Supplementary file 1 [file pathogens-14-00556-s001.zip › pathogens-3620602-supplementary.pdf]

Table S1. Neutralizing sensitivity of CBJC437 pseudoviruses to PGT135.

Pseudoviruses	IC50 (μg/mL)
CBJC437-200507-1	0.2
CBJC437-200507-2	1.5
CBJC437-200507-4	0.7
CBJC437-200507-5	2.9
CBJC437-200507-6	1.6
CBJC437-200507-7	0.7
CBJC437-200507-8	1.7
CBJC437-200507-9	1.4
CBJC437-200507-10	0.7
CBJC437-200507-11	0.2
CBJC437-200510-1	0.5
CBJC437-200510-3	0.5
CBJC437-200510-4	0.2
CBJC437-200510-5	0.3
CBJC437-200510-9	1.2
CBJC437-200510-10	0.8
CBJC437-200803-1	0.2
CBJC437-200803-2	0.1
CBJC437-200803-3	0.1
CBJC437-200803-4	0.4
CBJC437-200803-5	0.2
CBJC437-200912-2	0.2
CBJC437-200912-5	0.2
CBJC437-200912-6	0.3
CBJC437-200912-7	1.0
CBJC437-200912-8	0.6

CBJC437: sample ID.

200507: Indicates collection date (July 2005), and the same applies to 200510, 200803, and 200912.

Table S2. The neutralizing sensitivity of CBJC437 mutant pseudoviruses to PGT135.

CBJC437 mutant pseudoviruses	IC50 (μg/mL)
CBJC437-200507-8 (N234A)	0.7
CBJC437-200507-8 (N405A)	0.1
CBJC437-200507-8 (N411A)	0.5
CBJC437-200507-9 (N234A)	0.3
CBJC437-200507-9 (N405A)	0.1
CBJC437-200507-9 (N411A)	0.7
CBJC437-200507-10 (N234A)	2.9
CBJC437-200507-10 (N405A)	<0.1
CBJC437-200507-10 (N411A)	2.9
CBJC437-200507-11 (N234A)	0.7

CBJC437-200507-11 (N405A)	0.1
CBJC437-200507-11 (N411A)	0.4

Table S3. The neutralizing sensitivity of CBJC515 mutant pseudoviruses to PGT135.

CBJC515 mutant pseudoviruses	IC50 (μg/mL)
CBJC515 -2006-4 (N398A)	7.2
CBJC515 -2008-8 (N230A)	>10
CBJC515 -2008-10 (N230A)	>10
CBJC515 -2008-10 (N398A)	>10
CBJC515 -2008-11 (N230A)	>10
CBJC515 -2008-13 (N398A)	6.9
CBJC515 -2008-9 (N611A)	2.6
CBJC515 -2008-1 (N230A)	>10
CBJC515 -2008-1 (N611A)	>10
CBJC515 -2008-12 (N611A)	-

'>10' indicates the pseudovirus resistance to PGT135.

'-' denotes non-functional strain that failed to produce infectious pseudovirus.

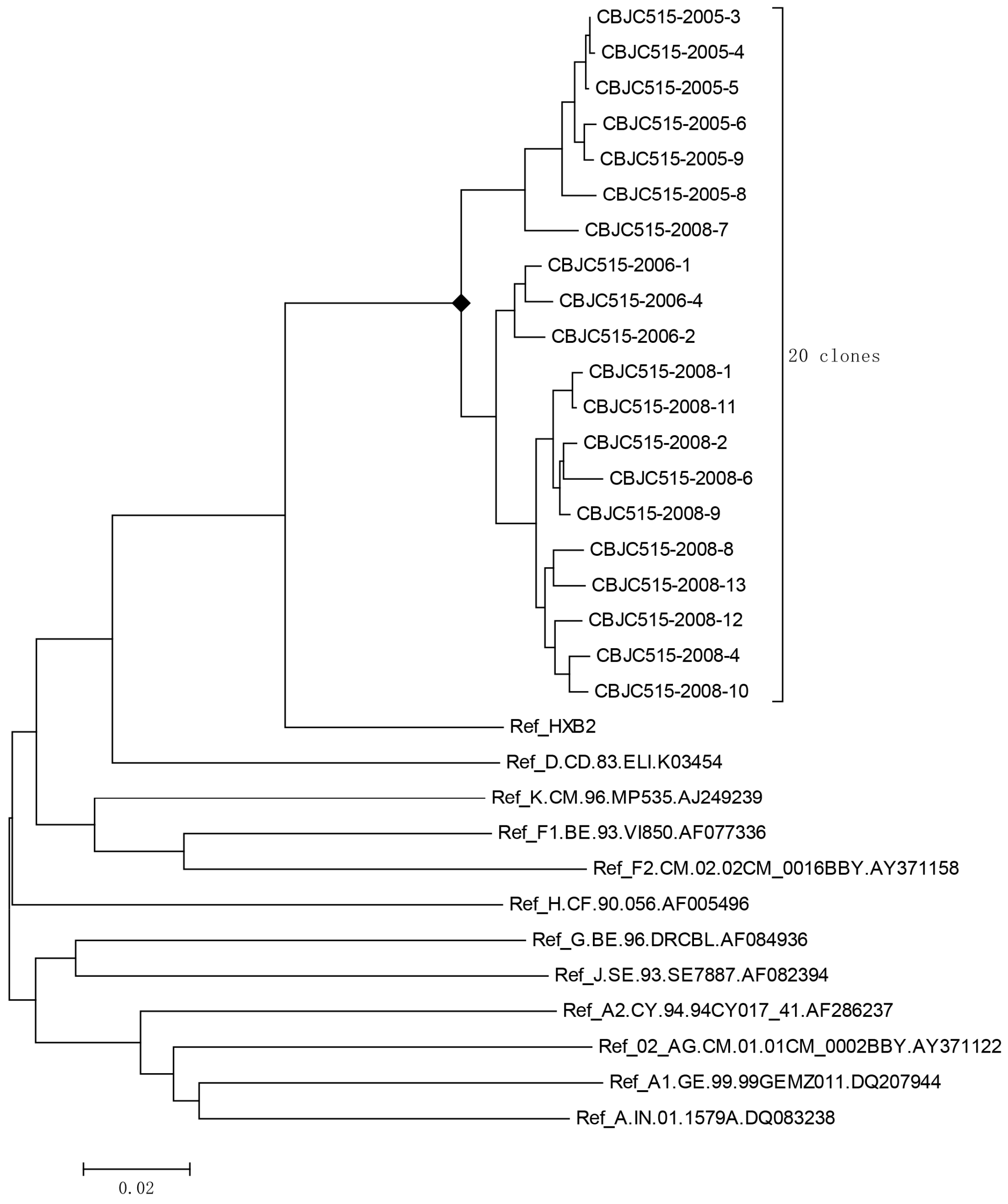

Figure S1. The neighbor-joining phylogenetic tree showing the placement of 20 clones within global HIV reference strains.

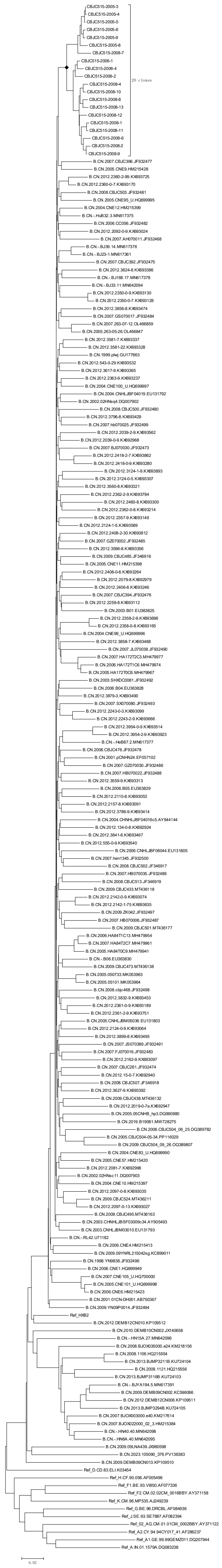
20 clones
0.02

Figure S2. The neighbor-joining phylogenetic tree illustrating the distribution of 20 clones within the Chinese subtype B reference dataset.
